# A self-feedback network based on liquid chromatography-quadrupole-time of flight mass spectrometry for system identification of β-carboline alkaloids in *Picrasma quassioides*

**DOI:** 10.1038/s41598-017-13106-8

**Published:** 2017-10-23

**Authors:** Yuanyuan Shi, Ruoqi Wang, Xiaoyu Zhu, Dongge Xu, Wenyuan Liu, Feng Feng

**Affiliations:** 1 Department of Pharmaceutical Analysis, China Pharmaceutical University, Nanjing, 210009 China; 2 Key Laboratory on Protein Chemistry and Structural Biology, China Pharmaceutical University, Nanjing, 210009 China; 3 Key Laboratory of Biomedical Functional Materials, China Pharmaceutical University, Nanjing, 211198 China

## Abstract

Profiling chemical components in herbs by mass spectrometry is a challenging work because of the lack of standard compounds, especially for position isomers. This paper provides a strategy based on a self-feedback network of mass spectra (MS) data to identify chemical constituents in herbs by liquid chromatography-quadrupole-time of flight mass spectrometry without compound standards. Components sharing same skeleton were screened and all ions were classified into a database. All candidates were connected by the selected bridging ions to establish a primary MS network. Benefited from such a network, it is feasible to characterize sequentially the structures of all diagnostic ions and candidates once single component has been de novo identified. Taking *Picrasma quassioides* as an example, the primary network of β-carbolines was established with 65 ions (selected from 76 β-carbolines), each of which appeared at least in four compounds. Once an alkaloid has been identified, its logical ions could feedback into primary network to build pathways with other unknown compounds. Moreover, the position of the substituent groups could be deduced through the secondary metabolic pathways of alkaloids (plant secondary metabolism). The network therefore can be utilized for identification of unknown compounds and even their position isomers.

## Introduction

Herbal medicines have been used for the prevention and treatment of various diseases for thousands of years in China and their curative effects were also confirmed by modern pharmacological researches. Moreover, methods for profiling multiple chemical components in herbal medicines are of great concerns because these complex components are the basis for their therapeutic effects and quality control. Currently, high performance liquid chromatography coupling tandem mass spectrometry (LC-MS^n^) has been considered as the most sensitive high-throughput technique for profiling complex chemical matrices^1^. Usually, reference compounds and/or their reported mass spectrometric data are necessary and serve as aids for identification of components by LC-MS^n^ ^2–4^. However, obtaining the reference compounds is a challenge and time consuming work. Moreover, natural products usually share with isomers in herbs. Therefore, it is challenging to distinguish chemical components by mass spectrometry because of the difficulty to determine the exact structures when multiple isomers are available.

Up to now, numerous efforts have been made to identify compounds more rapidly and accurately without or with few standards. Among them, mass defect filter (MDF) is a popular data processing tool applied for distinguishing metabolites in biosamples and chemical constituents in traditional Chinese medicines (TCMs)^5,13^. It could segregate and detect the compounds by imposing preset criteria around the mass defects and selected core substructures. In general, structural analogues in herb usually shared with a similar core substructure and characterized by various chemical groups including hydroxyls, formyls, methoxys, methyls, and glycosyls. Thus, the similar core substructure with significantly lower molecular mass could be utilized as MDF template compound. And the chemical groups provided the changes and limited ranges. Once the preset filtering setting applied, the characteristic compounds could be extracted and the heterogeneous ions could be removed^5,13^. An approach of liquid chromatography-hybrid ion trap time-of-flight mass spectrometry (LC-IT-TOF-MS) with MDF technique was developed to extract and classify peaks according to the structure types. By using this approach, 50 ophiopogonins and 27 ophiopogonones were structurally classified and determined from the extract of *Ophiopogon japonicus* ^5^. Also, a study developed an improved strategy using MDF in combination with LC-IT-TOF-MS analysis and theoretical calculations for identification and structural characterisation of dihydroindole-type alkaloids in processed *Semen Strychni* extracts. Twenty-four dihydroindole-type alkaloids, including four that were previously not described, were tentatively identified^14^. Moreover, ion fragment pathways were applied for identification. Based on LC-IT-TOF-MS, a novel and generally applicable approach to identifying nontarget components from herbal preparations was developed. By classified peaks into families based on the exact same fragment ions (mass error <5 mDa) and connected all families by peaks that presented in two or more families, this method was successfully applied to identify 43 compounds from an herbal preparation^6^. In our laboratory, 18 tetracyclic monoterpenoid oxindole alkaloids in *Uncaria rhynchophylla* were determined by analyzing their LC-MS^2^ cleavage pathways and metabolic relationships^7^. However, all of the above approaches are restricted due to the needs of the predefined ion fragments of known compounds summarized from standards and/or literatures. Because of the scarcity of the standard compounds for the majority of herbal medicines and natural products, it is urgent to develop a new strategy to identify compounds without standards.

*Picrasma quassioides* (D. Don) Benn. (Fam. Simaroubaceae), called Kumu in Chinese, is one of TCMs for the treatment of swollen sore throat, diarrhea and dysentery, eczema, sore and deep-rooted boil, and bite wound of insect or snake, or as a gastrointestinal vermifuge agent^8,9^. It has been listed in all versions of Chinese Pharmacopeia and has been used singly (Kumu Injection) or as an ingredient for many Chinese herbal preparations. Previous investigation showed that alkaloids, including β-carboline, canthinone and the dimers of them, were the principal active components in *P. quassioides* ^10,11^. A high performance liquid chromatography (HPLC) fingerprint has been constructed from the extract of *P. quassioides*. Only 7 alkaloids of 27 peaks were identified^12^. Also, in our laboratory, a method of total ion chromatogram combined with chemometrics and MDF was established for the prediction of antitumor active ingredients in *P. quassioides* samples. A total of 17 constituents were predicted as the potential antitumor active compounds, and only 12 of which were identified^13^. Because there are too many isomers and not enough standards could be available, it is difficult to identify and/or even predict the druggability of the alkaloids in *P. quassioides*. Therefore, it is a challenging work to develop a methodology for comprehensive identification of such components with numerous isomers in the absence of standards.

Herein, we described an efficient and practicable strategy to identify alkaloids, including structural analogues such as isomers, in *P. quassioides* based on developed self-feedback enhanced network by quadrupole-time of flight mass spectrometry (LC-Q-TOF MS). This strategy was aimed to establish a primary network with the most common fragment ions (the number of times the ion appeared ≥4) of alkaloids by MDF technique. Once a single alkaloid has been identified, its fragment ions presented in the primary network could be connected by the cleavage pathways, while some logical ions (the maximum tolerance of mass error <5 mDa with a rational predicted molecular formula) which were not presented in the primary network could be feedback to establish an enhanced network. The identifying capability of this network will become ever more powerful with more and more ions being feedback to it from the identified compounds. Based on the chemical information obtained from literatures, the structures of a particular type of constituents could be easily identified by this strategy without standards. It is useful for the quality control and component identification of various natural products, such as herbal medicines, preparations and other biological samples.

## Results and Discussion

**Figure 1:**
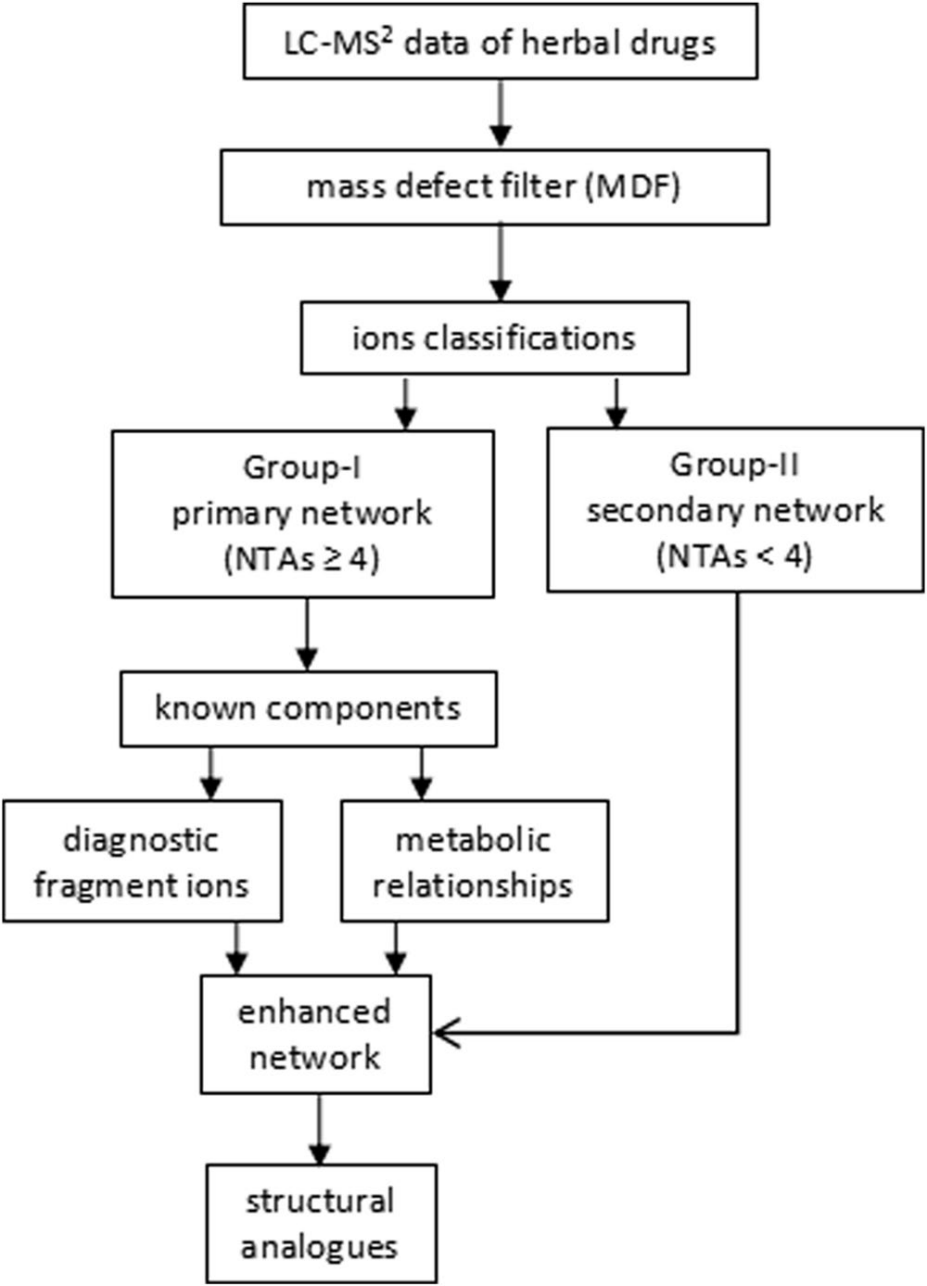
Workflow for the component identification with the strategy of self-feedback network.

The workflow of this strategy was described in the method section and shown in Fig. 1.

### Extraction of alkaloid candidates by MDF

**Figure 2:**
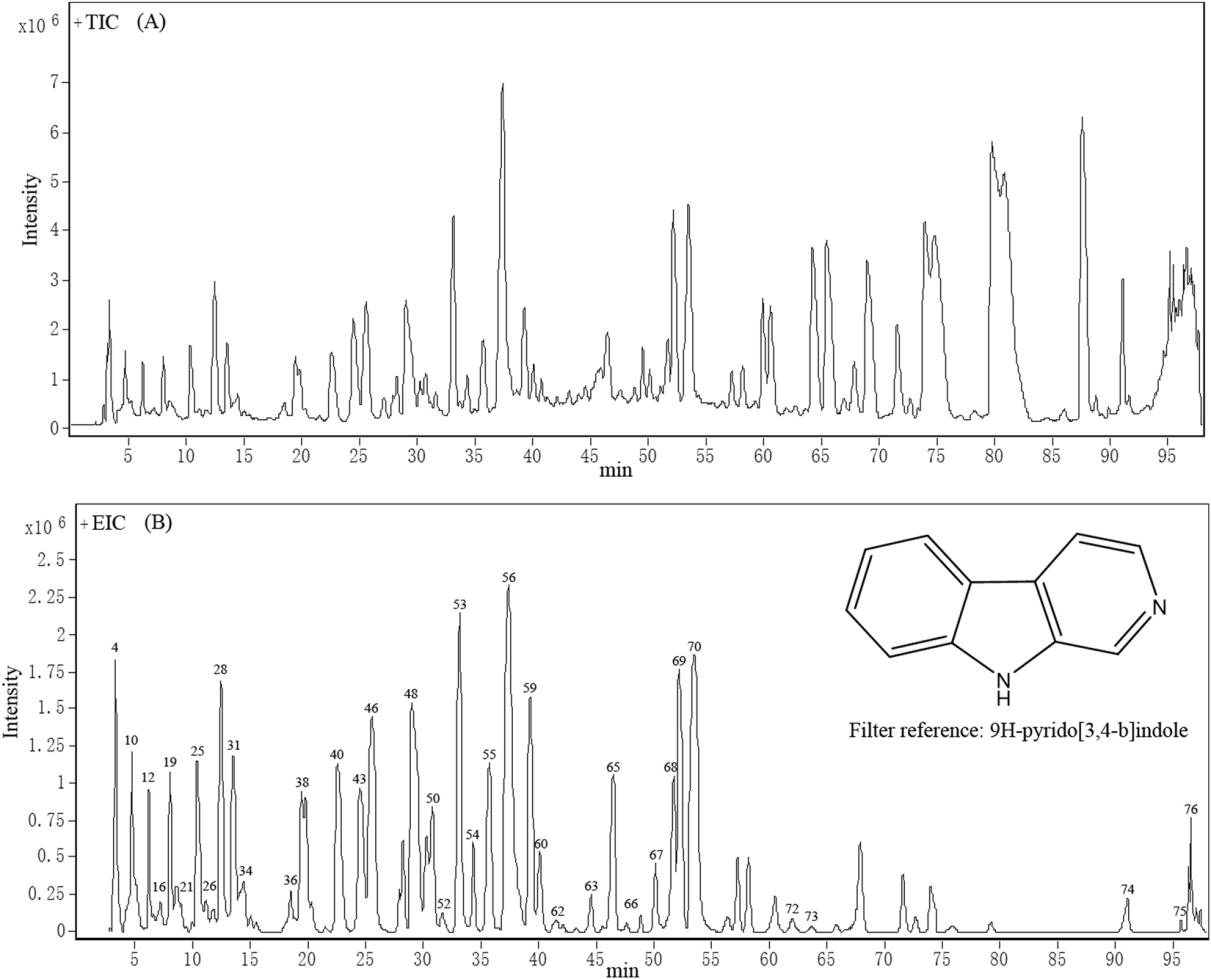
Total ion chromatogram obtained by LC-Q-TOF MS (**A**) and corresponding filtered chromatogram of single β-carboline alkaloids by mass defect filter (**B**).

The extract of *P. quassioides* was detected by LC-Q-TOF MS, and the total ion chromatogram (TIC) was shown in Fig. 2A. Under the selected conditions, most of the peaks were well separated with high resolution and good sensitivity. However, the alkaloids contained in *P. quassioides* were the major active components. In order to reduce interferences of other ions in matrix, the potential alkaloids, divided into single (β-carbolines and canthinones) and dimer alkaloids, were extracted by MDF technique.

For the single alkaloids, the mass defects of 9*H*-pyrido[3,4-*b*]indole (C_11_H_8_N_2_) (filter reference) and all its derivatives with various chemical substituents were summarized (Supplementary Table S1). Based on the information of Supplementary Table S1, the minimum and maximum values of mass defects were calculated as 0.0378 and 0.1790 Da, corresponding to the formula of C_11_H_6_N_2_O_3_ and C_18_H_23_N_3_O_2_, respectively. Therefore, the filter was set as C_14.5_H_14.5_N_2.5_O_2.5_ ± 70.6 mDa over the mass range of 160–400 Da. The filtered chromatogram was shown in Fig. 2B and its noise level (2.5 × 10 e^6^ counts per second, cps) was 36% of that of the original chromatogram (7 × 10 e^6^ cps) (Fig. 2A). After excluding the irrelevant ions by MDF, a total of 76 single candidates (Table 1) were detected in the filtered TIC profiles.

**Table 1:** Identified single alkaloids from the extract of *P. quassioides*.

No.	Rt (min)	[M + H]^+^ (m/z)	Tolerance (mDa)	Predicted formula	Assignment	Remark
1	3.144	215.0800	1.48	C_12_H_10_N_2_O_2_	unknown	/
2	3.312	183.0903	1.33	C_12_H_10_N_2_	4-methyl-β-carboline	IE, P
3	3.324	213.1008	1.42	C_13_H_12_N_2_O	1-(1-hydroxyl)-ethyl-β-carboline	IE, P
4	3.339	199.0850	1.54	C_12_H_10_N_2_O	1-hydroxymethyl-β-carboline	IP, 15
5	3.403	211.0852	1.34	C_13_H_10_N_2_O	1-ethenyl-3-hydroxyl-β-carboline	IE, P
6	3.418	241.0958	1.33	C_14_H_12_N_2_O_2_	β-carboline-1-propanoic acid	IP, 16
7	3.443	229.0595	1.31	C_12_H_8_N_2_O_3_	unknown	/
8	3.467	259.1063	1.39	C_14_H_14_N_2_O_3_	1-(1,2-dihydroxyl)-ethyl-4-methoxyl-β-carboline	IP, 17
9	4.500	213.1008	1.47	C_13_H_12_N_2_O	1-hydroxymethyl-4-methyl-β-carboline	IE, N
10	4.731	229.0594	1.33	C_12_H_8_N_2_O_3_	7-hydroxyl-β-carboline-1-carboxylic acid	IP, 16
11	6.119	183.0903	1.41	C_12_H_10_N_2_	1-methyl-β-carboline	IP, P,
12	6.239	199.0852	1.36	C_12_H_10_N_2_O	4-hydroxymethyl-β-carboline	IE, P
13	6.654	211.0851	1.53	C_13_H_10_N_2_O	1-ethenyl-8-hydroxyl-β-carboline	IE, P
14	7.004	267.0750	1.44	C_15_H_10_N_2_O_3_	3-methyl-4-hydroxylcanthin-5,6-dione	IP
15	7.024	255.1113	1.41	C_15_H_14_N_2_O_2_	1-methoxypropionyl-β-carboline	IP,
16	7.211	257.0907	1.13	C_14_H_12_N_2_O_3_	β-carboline-1-(2′-hydroxyl)-propanoic acid	IP,
17	7.289	255.1117	1.34	C_15_H_14_N_2_O_2_	1-(2′,3′-dihydroxyl-2)-butenyl-β-carboline	IE, N
18	7.917	183.0903	1.39	C_12_H_10_N_2_	2-methyl-β-carboline	IE, P
19	8.563	241.0958	1.30	C_14_H_12_N_2_O_2_	1-methenyl-4-methoxyl-β-carboline	IP,
20	8.077	213.1008	1.42	C_13_H_12_N_2_O	1-(2-hydroxyl)-ethyl-β-carboline	IE, P
21	8.238	229.0594	1.22	C_12_H_8_N_2_O_3_	unknown	/
22	8.567	227.0803	1.40	C_13_H_10_N_2_O_2_	1-(1′,2′-dihydroxyl)-ethenyl-β-carboline	IE, N
23	8.985	213.1008	1.39	C_13_H_12_N_2_O	1-methoxymethyl-β-carboline	IE, P,
24	10.024	195.0903	1.47	C_13_H_10_N_2_	1-ethenyl-β-carboline	IP, P
25	10.431	213.0644	1.27	C_12_H_8_N_2_O_2_	β-carboline-1-carboxylic acid	IP, St
26	11.158	281.0920	1.47	C_16_H_12_N_2_O_4_	3-methyl-4-methoxylcanthin-5,6-dione	IP,
27	11.873	257.0908	1.42	C_14_H_12_N_2_O_3_	7-hydroxyl-β-carboline-1-propanoic acid	IP, P,
28	12.446	251.0801	1.48	C_15_H_10_N_2_O_2_	3-methylcanthin-5,6-dione	IP, St
29	13.169	223.0851	1.46	C_14_H_10_N_2_O	4,5-dihydrocanthin-6-one	IP, P,
30	13.530	229.0957	1.49	C_13_H_12_N_2_O_2_	1-(1’-hydroxyl)-ethyl-4-hydroxyl-β-carboline	IE, N
31	13.545	241.0958	1.40	C_14_H_12_N_2_O_2_	1-ethenyl-4-methoxyl-8-hydroxyl-β-carboline	IP,
32	14.013	289.1170	1.33	C_15_H_16_N_2_O_4_	β-carboline-1-(1,2-dihydroxyl)-propanoic acid	IP
33	14.139	229.0593	1.47	C_12_H_8_N_2_O_3_	unknown	/
34	14.436	243.1113	1.53	C_14_H_14_N_2_O_2_	1-ethyl-4-methoxyl-8-hydroxyl-β-carboline	IP,
35	17.987	213.1004	1.84	C_13_H_12_N_2_O	1-methyl-4-methoxyl-β-carboline	IP
36	18.505	227.1160	1.90	C_14_H_14_N_2_O	1-ethyl-9-methoxyl-β-carboline	IE, P
37	19.023	269.1269	1.53	C_16_H_16_N_2_O_2_	6-isopropyl-β-carboline-1-acetic acid	IE, P
38	19.861	225.1007	1.50	C_14_H_12_N_2_O	1-ethenyl-9-methoxyl-β-carboline	IP, P,
39	22.428	237.0643	1.50	C_14_H_8_N_2_O_2_	11-hydroxylcanthin-6-one	IP
40	22.617	225.1009	1.35	C_14_H_12_N_2_O	1-ethenyl-4-methoxyl-β-carboline	IP,
41	23.021	221.0695	1.45	C_14_H_8_N_2_O	canthin-4-one	IE, P, St
42	24.444	243.1113	1.51	C_14_H_14_N_2_O_2_	1-hydroxyethyl-4-methoxyl-β-carboline	IE, P,
43	24.555	227.1161	1.87	C_14_H_14_N_2_O	1-ethyl-4-methoxyl-β-carboline	IP,
44	24.574	243.0750	1.46	C_13_H_10_N_2_O_3_	4-methoxyl-β-carboline-1-carboxylic acid	IP
45	25.151	301.1170	1.29	C_16_H_16_N_2_O_4_	4,8-dimethoxyl-β-carboline-1-(1′-hydroxyl)-propanaldehyde	IE, N
46	25.580	227.0804	1.08	C_13_H_10_N_2_O_2_	1-methoxymethenyl-β-carboline	IP, 16,
47	29.131	255.1116	1.23	C_15_H_14_N_2_O_2_	1-ethenyl-4,8-dimethoxyl-β-carboline	IP,
48	29.646	237.0646	1.30	C_14_H_8_N_2_O_2_	8-hydroxylcanthin-6-one	IP,
49	30.267	243.0753	1.08	C_13_H_10_N_2_O_3_	1-methoxymethenyl-4-hydroxyl-β-carboline	IE, N
50	30.759	257.1272	1.24	C_15_H_16_N_2_O_2_	1-ethoxymethyl-4-methoxyl-β-carboline	IE, N
51	31.109	297.0858	1.21	C_16_H_12_N_2_O_4_	6-methoxyl-canthin-5-one-4-methanoic acid	IE, N
52	31.670	185.0698	1.19	C_11_H_8_N_2_O	3-hydroxyl-β-carboline	IP,
53	34.323	297.0859	1.04	C_16_H_12_N_2_O_4_	4,9-dimethoxyl-5-hydroxylcanthin-6-one	IE, N
54	34.349	257.1271	1.35	C_15_H_16_N_2_O_2_	1-ethyl-4,8-dimethoxyl-β-carboline	IP,
55	35.711	241.0963	0.99	C_14_H_12_N_2_O_2_	1-ethoxymethenyl-β-carboline	IP,
56	38.103	267.0757	0.68	C_15_H_10_N_2_O_3_	4-methoxyl-5-hydroxylcanthin-6-one	IP, St
57	38.391	273.0862	0.80	C_14_H_12_N_2_O_4_	1-methoxymethenyl-4-methoxyl-8-hydroxyl-β-carboline	IP
58	39.182	301.1537	1.01	C_17_H_20_N_2_O_3_	1-ethoxyethyl-4,8-dimethoxyl-β-carboline	IE, N
59	39.270	255.1119	0.87	C_15_H_14_N_2_O_2_	1-ethenyl-4,9-dimethoxyl-β-carboline	IP,
60	40.064	251.0806	0.88	C_15_H_10_N_2_O_2_	3-methylcanthin-2,6-dione	IP
61	40.248	215.0805	0.98	C_12_H_10_N_2_O_2_	4-hydroxyl-1-methoxyl-β-carboline	IE, N
62	41.301	237.0648	1.10	C_14_H8N_2_O_2_	2-hydroxyl-canthin-6-one	IE, P,
63	44.384	227.0804	1.10	C_13_H_10_N_2_O_2_	8-hydroxyl-β-carboline-1-acetaldehyde	IE, N
64	44.560	245.0911	0.98	C_13_H_12_N_2_O_3_	3-ethoxyl-1,2,3,4-tetrahydro-1,4-dioxo-β-carboline	IE, N
65	46.429	221.0699	0.99	C_14_H_8_N_2_O	canthin-6-one	IP
66	47.634	291.0756	0.84	C_17_H_10_N_2_O_3_	unknown	/
67	50.798	241.0970	0.19	C_14_H_12_N_2_O_2_	1-ethenyl-4-methoxyl-6-hydroxyl-β-carboline	IE, P, 24,
68	51.670	257.0910	1.08	C_14_H_12_N_2_O_3_	4-methoxyl-β-carboline-1-acetic acid	IE, N
69	51.964	241.0968	0.36	C_14_H_12_N_2_O_2_	1-propenyl-4,8-dihydroxyl-β-carboline	IE,N
70	53.489	281.0912	0.85	C_16_H_12_N_2_O_3_	4,5-dimethoxylcanthin-6-one	IP, St
71	54.361	257.0911	1.00	C_14_H_12_N_2_O_3_	1-acetyl-4-methoxyl-8-hydroxyl-β-carboline	IE, N
72	62.191	271.1065	1.20	C_15_H_14_N_2_O_3_	4-methoxyl-10-hydroxyl-11-methoxyl-cyclo-β-carboline	IE, N
73	63.631	287.1015	1.09	C_15_H_14_N_2_O_4_	1-(1′,2′-dihydroxyl)-ethyl-4,8-dimethoxyl-β-carboline	IE, N
74	90.855	271.1076	0.14	C_15_H_14_N_2_O_3_	1-ethanoyl-4,8-dimethoxyl-β-carboline	IE, N
75	95.643	255.1130	−0.16	C_15_H_14_N_2_O_2_	1-(2′-hydroxyl)-propenyl-4-methoxyl-β-carboline	IE, N
76	97.342	271.108	−0.26	C_15_H_14_N_2_ O_3_	1-((2′S/R)−2′,3′-dihydroxyl)−4-methyl-β-carboline (optical isomer)	IE, P
Kx1	5.501	245.0539	1.74	C_12_H_8_N_2_O_4_	8-methoxyl-1,2,3,4-Tetrahydro-1,3,4-trioxo-β-carboline	IE
Kx2	17.278	283.0695	1.59	C_15_H_10_N_2_O_4_	4,10-dihydroxyl-5-methoxylcanthin-6-one	IE,
Kx3	56.003	211.0857	0.85	C_13_H_10_N_2_O	1-ethanoyl-β-carboline	IE, St

IE: identified by the enhanced network, IP: identified by the primary network, N: the new structure; P: the structure found in *P. quassioides* for the first time, St: the identified structure was validated by standard, 15–31: the identified structure was validated by the information of indicated literature.

### Establishment of the primary network

Diagnostic ion pathways (cleavage pathway connected by diagnostic fragment ions (DFIs)) are useful in LC-MS^n^ analysis and have been widely applied for the rapid identification of compounds in various studies^2–4^. Generally, the way to construct ion cleavage pathways was based on chemical standards containing same carbon skeletons or substructures, from which the same fragment ions (i.e., DFIs) can be produced. Lack of enough chemical standards for selecting reasonable DFIs limits the application of ion cleavage pathways to identify the chemical constituents in herbal productions. Herein, a strategy to select reasonable DFIs with a primary network of ion fragments rather than chemical standards was proposed.

Based on the Q-TOF MS analysis, the 76 single alkaloid candidates gave a total of 231 MS^2^ ion fragments with the maximum tolerance of mass error less than 5 mDa. They could be divided into two groups: Group-I with 79 ions (the number of times the ion appeared (NTAs) ≥4) and Group-II with 152 ions (NTAs <4). Among Group-I, 65 ions which had logical losses of molecular weight and corresponded to a certain chemical formula were selected to establish the primary network (Supplementary Table S2). The selection of NTAs was able to include most of the alkaloid candidates with the least fragment ions to facilitate the compound identification.

### Identification of alkaloid candidates by primary network

**Figure 3:**
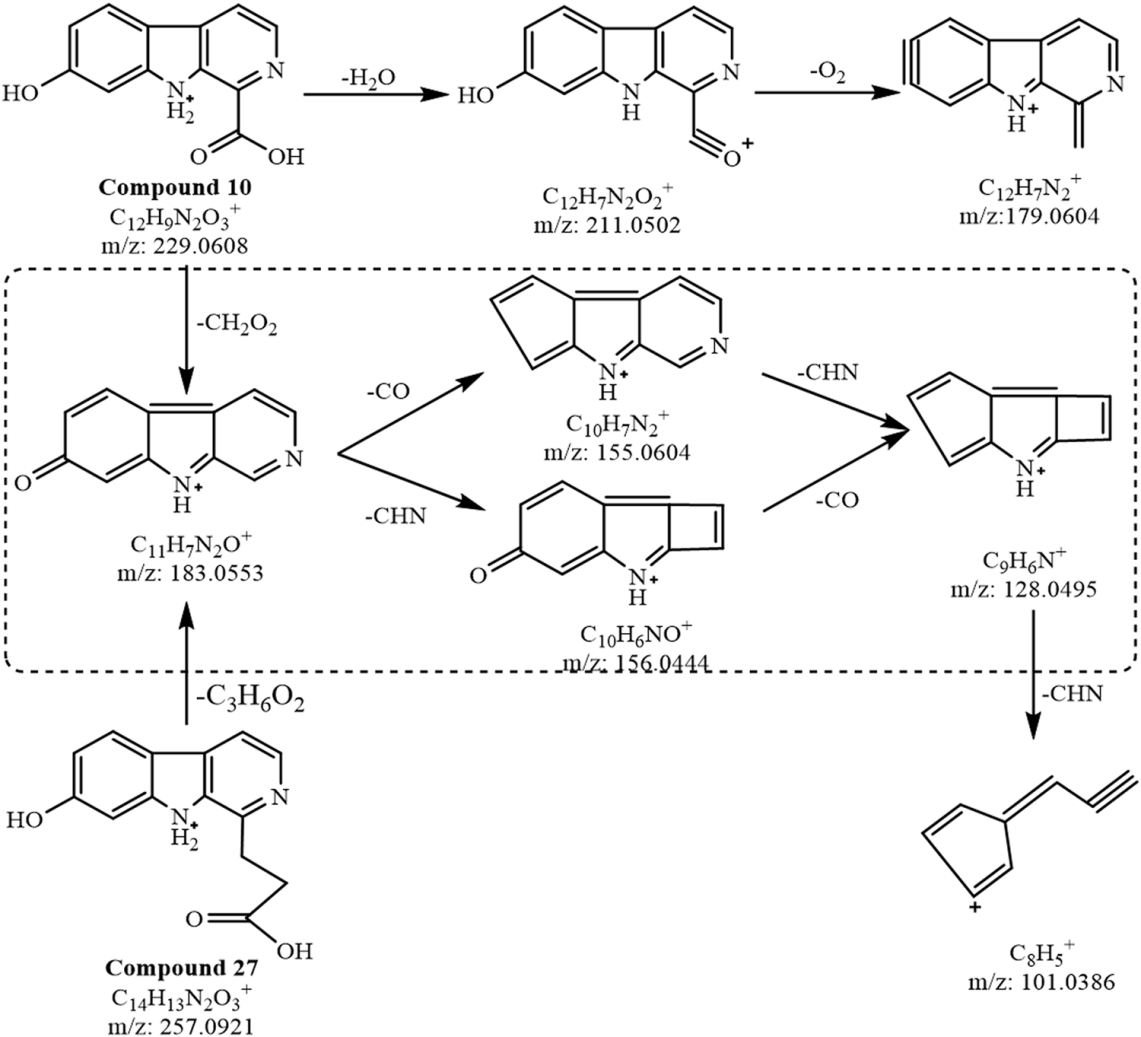
Cleavage pathways of compounds **10** and **27**.

**Figure 4:**
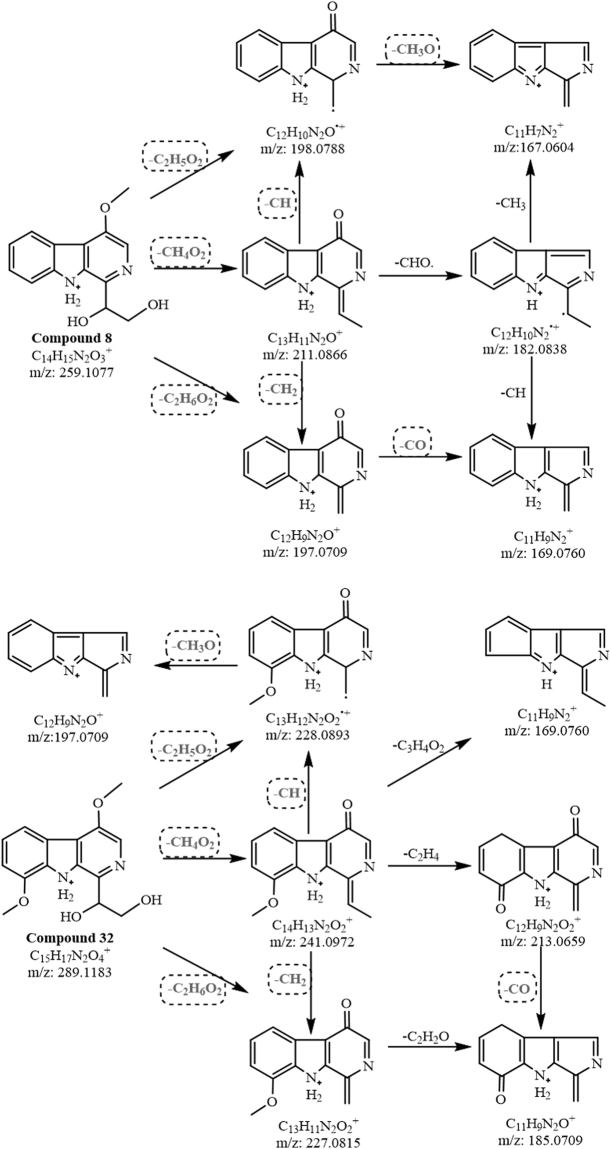
Cleavage pathways of compounds **8** and **32**.

The single alkaloid candidates captured by MDF were rearranged according to the number of isomers (including detected and reported) (Table 2). The candidates without isomers can be identified easily by the MS and/or MS^2^ ions with reported information. For example, compounds **8** and **32**, two of single alkaloids with [M + H]^+^ *m/z* at 259 and 289, were unambiguously identified by the precise relative molecular mass, respectively. Sometimes, although a compound has more than two reported isomers, its structure is also easily identified by the precise relative molecular mass and the MS^2^ ion fragments inferred from the reported isomers, such as compounds **10**, **52** and **57** ([M + H]^+^ *m/z* at 229, 185 and 273). After the identification of the above compounds, the MS^2^ ion fragments and their cleavage pathways of these compounds can be applied to extrapolate the compounds with the same ion fragments. Generally, the more the same ion fragments, the more the structure is similar. For example, there were 6 isomers at [M + H]^+^ *m/z* 257, but only compound **27** shared 4 of the same ion fragments with compound **10** at *m/z* 128.0495, 155.0604, 156.0444, 183.0553 in the primary network (Supplementary Table S2). Thus, compound **27** can be easily identified through the diagnostic ion pathways built with the above four ion fragments (Fig. 3). Besides, the metabolic relationship of two compounds in the plant is also useful for the structure identification. For example, compound **32**, having one more methoxyl at C-8 than compound **8**, can be considered as a metabolite of compound **8**. Therefore, the cleavage patterns of compounds **8** and **32** were very similar for losing seven identical neutral fragments (-C_2_H_5_O_2_, -CH_4_O_2_, -C_2_H_6_O_2_, -CH, -CH_2_, -CH_3_O and -CO) at the corresponding same positions (Fig. 4). This suggested that the structures, especially the position of the substituent group, of the compounds which showed the similar cleavage patterns (i.e., with the same neutral losses) can be deduced through their metabolic pathways.

**Table 2:** Detected and reported isomers of β-carboline alkaloids in *P. quassioides*.

[M + H]^+^ (m/z)	Detected Compound No.	Amount of isomer detected/eported
183	2, 11, 18	3/0
185	52	1/2
195	24	1/0
199	4, 12	2/2
211	5, 13	2/1
213	3, 9, 20, 23, 25, 35	6/3
215	1, 61	2/1
221	41, 65	2/1
223	29	1/0
225	38, 40	2/1
227	22, 36, 43, 46, 63	5/3
229	7, 10, 21, 30, 33	5/2
237	39, 48, 62	3/2
241	6, 19, 31, 55, 67, 69	6/4
243	34, 42, 44, 49	4/3
245	64	1/1
251	28, 60	2/3
255	15, 17, 47, 59, 75	5/3
257	16, 27, 50, 54, 68, 71	6/2
259	8	1/1
267	14, 56	2/3
269	37	1/0
271	72, 74, 76	3/0
273	57	1/4
281	26, 70	2/2
287	73	1/2
289	32	1/1
291	66	1/0
297	51, 53	2/0
301	45, 58	2/0

As a result, 31 of 76 single alkaloids (Table 1) were identified by the ion fragments in the primary network, combined with the structural or metabolic correlations between these compounds. The information of the ion fragments (including precise molecular mass, logical loss, percentage of relative abundance and tolerance) of the determined compounds can continually feed back to the primary network for correlating and analyzing more unidentified compounds.

### Enhancement of the network with feedback DFIs from the identified compounds

In order to identify the rest of the candidate alkaloids, the fragment ions in Group-II (NTAs <4) that involved into the cleavage pathways of the identified compounds were selected and fed back to the primary network. This network could be continually enhanced with more and more compounds being identified. The enhanced network was effective at discriminating the unidentified alkaloid analogues, especially the position isomers.

Compounds **6**, **19**, **31**, **55**, **67** and **69** are six isomers with the same molecular weight ([M + H]^+^ at *m/z* 241). As an example, their structures were distinguished by the enhanced network and the procedure was presented as follows. In the primary network, there were 10 ion fragments for compound **6**, 15 for **19**, 15 for **31**, 2 for **55**, 29 for **67** and 11 for **69**. They showed many same ion fragments because they are isomers. Among them, four isomers have been reported with the precise relative molecular mass of 240.2615 and a formula of C_14_H_12_N_2_O_2_ (Supplementary Table S1). By comparing the MS^2^ ion fragments, the structures of compounds **6, 19, 31, 55** could be assigned (Table 3). Compound **6** was easily determined as β-carboline-1-propanoic acid due to the ion fragments at *m/z* 223.0866 (C_14_H_11_N_2_O^+^, loss of H_2_O) and 195.0917 (C_13_H_11_N_2_^+^, loss of CH_2_O_2_). In addition to having two ion fragments (*m/z* 223.0866 and 195.0917) that were the same as compound **6**, compound **19** showed an ion fragment at *m/z* 226.0737 (C_13_H_10_N_2_O_2_^+^, loss of CH_3_) in the primary network and an ion fragment at *m/z* 199.0628 (C_12_H_9_NO_2_^+^, loss of C_2_H_4_N) in the secondary network (Group-II). Thus, compound **19** could be determined as 1-methenyl-4-methoxyl-β-carboline. Compound **31** also got an ion fragment at *m/z* 199.0628 (C_12_H_9_NO_2_^+^), which could continually lose CO and C_2_H_2_ (199.0628 → 171.0679 → 145.0522) or lose CHO (199.0628 → 170.0600). But differing from compound **19**, compound **31** had an ion fragment at *m/z* 225.0659 (C_13_H_9_N_2_O_2_^+^, loss of CH_4_) in the primary network and its structure could be deduced as 1-ethenyl-4-methoxyl-8-hydroxyl-β-carboline. Compound **55** only provided two ion fragments of 167.0604 (C_11_H_7_N_2_^+^, loss of C_3_H_6_O_2_, 70.26%) and 140.0495 (C_10_H_6_N^+^, loss of CHN from C_11_H_7_N_2_^+^, 29.74%) with high relative abundance, indicating that the structure was easily losing all the substituents to yield structurally stable residue. Therefore, compound **55** could be determined as 1-ethoxymethenyl-β-carboline. All the ion fragments and the cleavage pathways of the four compounds fed back to the enhanced network in order to identify the rest isomers and other compounds. Compound **67** shared 13, 7 and 4 ion fragments that were the same as compounds **31** (1-ethenyl-4-methoxyl-8-hydroxyl-β-carboline), **40** (1-ethenyl-4-methoxyl-β- carboline) and **24** (1-ethenyl-β-carboline), respectively (Supplementary Table S2). By compared with the above three compounds, compound **67** was determined as 1-ethenyl-4-methoxyl-6-hydroxyl-β-carboline. It is a known compound but found in *P. quassioides* for the first time, and its structure was confirmed by the MS^2^ ion fragments listed in Table 1. In the same way, compound **69** was identified as 1-propenyl-4,8-dihydroxyl-β-carboline, which shared 8 and 5 fragments that were the same as compounds **47** and **13**, respectively (Supplementary Table S2).

**Table 3:**
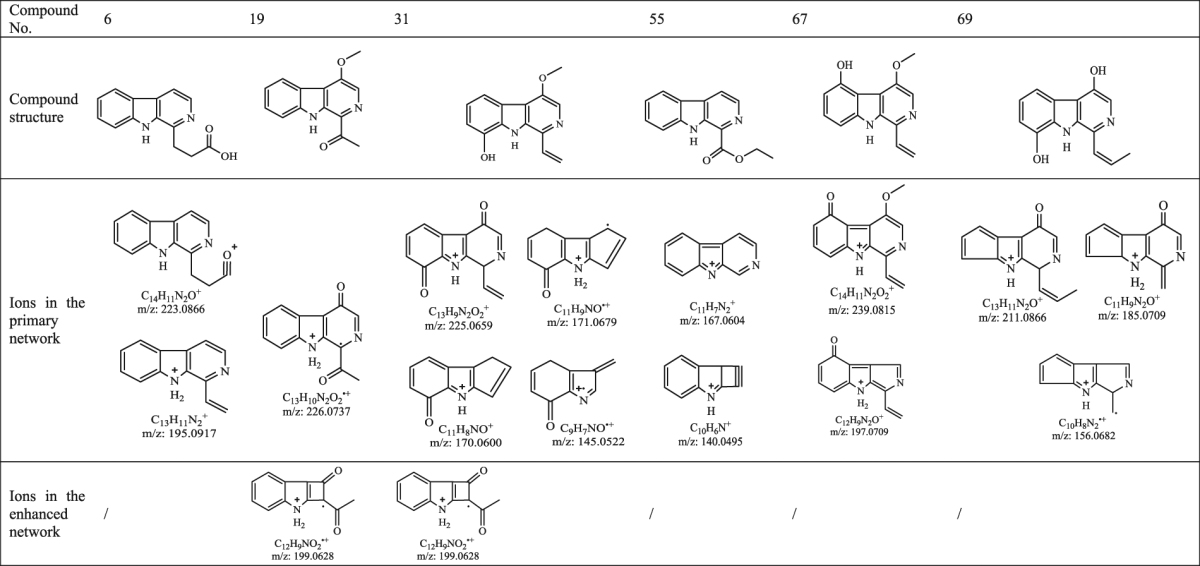
Comparison of the structures and fragment ions from compounds 6, 19, 31, 55, 67 and 69, six isomers with the same [M + H]^+^ at *m/z* 241.

By utilizing the enhanced network, 39 of the 45 unidentified single alkaloids were determined. As shown in Table 2, a total of 70 single alkaloids were identified by the primary and enhanced networks. Among them, 20 compounds were reported for the first time and 19 known compounds were detected in *P. quassioides* for the first time.

### Searching for missing alkaloids by the enhanced network

Ion fragments in the enhanced network can be used as DFIs to enlarge the screening ranges of alkaloids in *P. quassioides*. By using this strategy, three single alkaloids (**kx1, kx2, kx3**) that were not extracted by MDF technique were discovered and identified (Table 1). Compound **kx1** was screened due to four of the same ion fragments (171.0553, 144.0444, 143.0604, 116.0495) as compound **61** (4-hydroxyl-1-methoxyl-β-carboline) (Supplementary Fig. S1a), and it was determined as 8-methoxyl-1, 2, 3, 4-tetrahydro-1, 3, 4-trioxo-β-carboline. Compound **kx2** was discovered due to the ion fragment pathway from 184.0631 (C_11_H_8_N_2_O^+^) to 155.0604 (C_10_H_7_N_2_^+^) by losing CHO, and it was determined as 4, 10-dihydroxyl-5-methoxylcanthin-6-one (Supplementary Fig. S1b). Compound **kx3** showed 8 ion fragments (193.076, 192.0682, 169.076, 168.0682, 167.0604, 166.0651, 140.0495, 115.0542) belonging to the primary network (Supplementary Table S2). Compound **kx3**, with a [M + H]^+^ at *m/z* 211, was the isomers of compounds **5** (1-ethenyl-3-hydroxyl-β-carboline) and **13** (1-ethenyl-8-hydroxyl-β-carboline). By comparing their ions in the enhanced network, both compounds **5** and **13** had an ion fragment at *m/z* 131.0491 (C_9_H_7_O^+^) which compound **kx3** did not show (Supplementary Fig. S1c), indicating that compounds **5** and **13** had a cleavage pathway to loss two nitrogen but compound **kx3** did not. However, only compound **13** could lose a neutral fragment of CO (from 211.0866 to 183.0917). Thus, compound **kx3** was determined as 1-ethanoyl-β-carboline.

### Structure validation by chemical standards and literatures

As a result, totals of 79 single alkaloids were screened by the MDF technique and the enhanced network, and 73 of them were identified by the enhanced network (Table 1). Among them, the structures of **25**, **28**, **41**, **56**, **70** and **kx3** were confirmed by comparing with the standards, respectively. Moreover, the rationality of this strategy was also verified by comparing the ion fragments and the cleavage pathways of the standards with the corresponding identified compounds. Consulting with the literatures, the structures of 28 known alkaloids (Table 1) were also confirmed by analyzing their reasonable cleavage pathways and/or plant metabolic pathways. Among the identified compounds, 20 single alkaloids have not been reported before. Although we have revealed reasonable cleavage pathways to prove their possible structures, it is necessary to get more information to confirm the results due to the varied structures for a single fragment ion, which may lead to a wrong combination of the fragments. However, the case of wrong combinations would be not very likely based on the enhanced network, because a mismatch combination is easily observed and excluded by the cleavage pathways and the metabolic pathways.

Six single alkaloids (**1**, **7**, **21**, **33**, **66**, **76**) extracted by MDF technique were not successfully distinguished by this strategy. The main reason was the lack of enough fragment information. Among them, compound **76** was an alkaloid with two optical isomers which provide almost the same ion fragments and cannot be identified by this strategy.

In the same way, 16 of 35 dimer alkaloids were identified and 4 of them have not been reported before (Supplementary Fig. S2, Supplementary Table S3–S6).

## Conclusions

Alkaloids are one kind of secondary metabolites in natural products with significant biological and therapeutic activities. It is challenging to distinguish the position isomers of alkaloids because of the difficulty to determine the exact structure when multiple position isomers are available. In this paper, a strategy for determination of the structure of alkaloids has been developed based on self-feedback network of MS^2^ ions obtained from the LC-Q-TOF MS analysis, combining with the logical cleavage pathways and metabolic pathways. Taking *P. quassioides* as an example, 89 alkaloids, including 70 position isomers, had been successfully characterized by using this strategy. Moreover, 24 compounds had not been reported (new compounds), and 19 known compounds were detected in this herbal medicine for the first time. The results showed that this is an efficient and practical method to identify different kinds of secondary metabolites, especially some position isomers, in natural products.

## Materials and Methods

### Chemicals

Alkaloid standards of β-carboline-1-carboxylic acid, 3-methylcanthin-5,6-dione, 5-hydroxy-4-methoxycanthin-6-one, 4,5-dimethoxycanthin-6-one, 1-ethanoyl-β-carboline, canthin-4-one were isolated from *P. quassioides* in our laboratory and identified by MS^2^, ^1^H and ^13^C NMR spectral data. Their purities were determined to be more than 98% by HPLC method. HPLC grade acetonitrile was purchased from Shanghai Xingke Biochemistry Co., Ltd (Shanghai, China) and deionized water was prepared by a Milli-Q water purification system (Merck KGaA, Darmstadt, German). Other chemicals and solvents were of analytical grade and were obtained from Nanjing Chemical Reagent Co., Ltd. (Nanjing, China).

### Samples preparation

The 70% methanolic extract of *P. quassioides* was provided by Qingfeng Medical Investment Group (Jiangxi, China). The extract was accurately weighed and dissolved in methanol to prepare a sample solution containing 10 mg·mL^−1^. The reference compounds were separately dissolved in methanol to produce the reference solutions ranged from 0.365 to 0.115 mg·mL^−1^. Aliquots of 10 μL of samples and reference solutions were injected for LC-Q-TOF MS analysis after filtered through 0.45 μm millipore membrane.

### Chromatographic conditions

#### Mass spectrometric conditions

Mass spectra of test solutions were obtained from an Agilent 6520 Q-TOF spectrometry system (Agilent Corp., USA) equipped with an electrospray ionization interface. High purity nitrogen was used as the sheath gas and ultra high purity helium as the auxiliary gas. The sample was analyzed in positive ionization mode with the parameters as follows: drying gas flow at 8.0 L·min^−1^ and 325 °C, nebulizer 40 psi, sheath gas at 10 L·min^−1^ and 400 °C, capillary voltage at 3500 V, skimmer at 65 V, fragmentor voltage at 120 V. For MS/MS mode, the normalized collision energy was 35% with a resolution of 15000. Mass spectra were recorded in the range m/z 100–1700 for MS and 50–1700 for MS/MS. The activation time was 30 ms, and Agilent Mass Hunter Acquisition Software Ver. A.01.00 (Agilent Corp, USA) was used to control the parameters and to obtain the analytical data.

#### MDF Extraction and peak selection

For the single and dimer alkaloids contained in *P. quassioides*, 9H-pyrido[3,4-*b*]indole (C_11_H_8_N_2_) and 2 × 9H-pyrido[3,4-*b*]indole (C_22_H_16_N_4_) were selected as the filtering references, respectively, with calculated mass defects of 0.0687 Da and 0.1374 Da. The elemental compositions of target candidates ranged from 0 to 60 for carbon and hydrogen, from 0 to 8 for oxygen, from 2 to 4 for nitrogen and zero for other elements. Tolerance of predicted formula (mass error) less than 5 mDa. The candidate compounds were extracted according to the average element compositions of structural analogue ± mass defect tolerance (half width of mass defects range) by the qualitative analysis software Ver. B.04.00 (Agilent Corp., USA). And the relevant data of the candidate compounds including peak number, retention time, accurate mass, predicted chemical formula and the corresponding mass error were output.

#### Strategy for network construction and candidate alkaloid identification

For identification of the candidate alkaloids, the first step was to extract the rational MS fragment ions. All the fragment ions from all the target candidates with the exact molecular mass (the maximum tolerance of mass error <5 mDa) and predicted molecular formula was selected and rearranged according to the NTAs. Based on the NTAs, these ions were divided into two groups: Group-I with NTAs ≥4 and Group-II with NTAs <4. The ions in Group-I with logical losses of molecular weight corresponding to a definite chemical formula were selected to establish the primary network.

The secondary step was to determine the structures and the cleavage pathways of the ion fragments in the primary network. With the aids of the reported chemical information, the structures of known compounds could be easily identified. The fragment ions, which were obtained from these compounds and included in the primary network and can be linked by reasonable cleavage pathways, were utilized as DFIs. The DFIs can be utilized for determination of the same substructures.

The third step was to enhance the primary network with feedback DFIs. For the identified compounds, some fragment ions which can be linked by reasonable cleavage pathways were not occurred in Group-I but in Group-II. Such fragment ions, together with the cleavage pathways, could be fed back to the primary network to strengthen the identification function of the system.

After the identification of the known compounds, the unknown candidate alkaloids were mainly characterized by the enhanced network, combining with the cleavage pathways (including diagnostic central losses) and the metabolic relationships between the same type compounds. The new DFIs which were obtained from the identified alkaloids and included in Group-II could be continually fed back to the network.

## Electronic supplementary material

Supplementary information
