# Supplementary material for: A self-feedback network based on liquid chromatography-quadrupole-time of flight mass spectrometry for system identification of β-carboline alkaloids in Picrasma quassioides
Source: Sci Rep. 2017 Oct 23;7:13841. doi: 10.1038/s41598-017-13106-8 (PMC5653770; doi:10.1038/s41598-017-13106-8)

Supplementary information

All the information mentioned below (including supplementary tables, supplementary figures, supplementary figure legends and supplementary materials) were attached to the main manuscript.

The information of the main manuscript:

Title:

A self-feedback network based on liquid chromatography-quadrupole-time of flight mass spectrometry for system identification of β-carboline alkaloids in *Picrasma quassioides*

Authors information:

Yuanyuan Shi [a], Xiaoyu Zhu [a], Dongge Xu [a], Wenyuan Liu [a, b, *], Feng Feng [c,*]

[a] *Department of Pharmaceutical Analysis, China Pharmaceutical University, Nanjing 210009, China*

[b] *Key Laboratory on Protein Chemistry and Structural Biology, China Pharmaceutical University, Nanjing 210009, China*

[c] *Key Laboratory of Biomedical Functional Materials, China Pharmaceutical University, Nanjing 211198, China*

*** Corresponding author**

Professor Wenyuan Liu

Department of Pharmaceutical Analysis in China Pharmaceutical University, Tongjiaxiang 24, Nanjing 210009, China

Key Laboratory on Protein Chemistry and Structural Biology, China Pharmaceutical University, Nanjing 210009, China

Tel: +86 25 8327 1038

Fax: +86 25 8327 1038

Email: liuwenyuan8506@163.com

Professor Feng Feng

Key Laboratory of Biomedical Functional Materials, China Pharmaceutical University, Nanjing 211198, China

Tel: +86 25 8327 1038

Fax: +86 25 8327 1038

Email: fengsunlight@163.com

Table S1. Filter references for MDF: single β-carbolines and canthinones summarized from literatures

Compound	Molecular formula	Molecular weight	Formula change				Mass defect	Mass defect shift (Da)
			C-	H-	N-	O-		
Alkaloid standard	$C_{11}H_8N_2$	168.1980	/	/	/	/	0.0687	/
1-hydroxyl-β-carboline	$C_{11}H_8N_2$	184.1973	0	0	0	1	0.0637	-0.0050
1-methenyl-β-carboline	$C_{11}H_8N_2O$	196.2083	1	0	0	1	0.0637	-0.0050
1-hydroxymethyl-β-carboline	$C_{12}H_8N_2O$	198.2242	1	2	0	1	0.0637	-0.0050
1-methoxyl-β-carboline	$C_{12}H_{10}N_2O$	198.2242	1	2	0	1	0.0793	0.0106
1,6-dihydroxyl-β-carboline	$C_{12}H_{10}N_2O$	200.1967	0	0	0	2	0.0586	-0.0101
1-ethanoyl-β-carboline	$C_{11}H_8N_2O_2$	210.2352	2	2	0	1	0.0793	0.0106
1-methyl-4-methoxyl-β-carboline	$C_{13}H_{10}N_2O$	212.2511	2	4	0	1	0.0950	0.0263
1-methenyl-4-hydroxyl-β-carboline	$C_{13}H_{12}N_2O$	212.2077	1	0	0	2	0.0586	-0.0101
β-carboline-1-carboxylic acid	$C_{12}H_8N_2O_2$	212.2077	1	0	0	2	0.0586	-0.0101
1,2,3,4-tetrahydro-1,3,4-trioxo-β-carboline	$C_{13}H_{12}N_2O$	214.1802	0	-2	0	3	0.0378	-0.0309
canthin-6-one	$C_{11}H_6N_2O_3$	220.2303	0	0	0	0	0.0637	0.0000
1-ethenyl-4-methoxyl-β-carboline	$C_{14}H_8N_2O$	224.2621	3	4	0	1	0.0950	0.0263
1-methenyl-4-methoxyl-β-carboline	$C_{14}H_{12}N_2O$	226.2346	2	2	0	2	0.0742	0.0055
1-methoxymethenyl-β-carboline	$C_{13}H_{10}N_2O_2$	226.2346	2	2	0	2	0.0586	-0.0101
1-ethyl-4-methoxyl-β-carboline	$C_{13}H_{10}N_2O_2$	226.2780	3	6	0	1	0.1106	0.0419
4,8-dimethoxyl-β-carboline	$C_{14}H_{14}N_2O$	228.2505	2	4	0	2	0.0899	0.0212
11-hydroxylcanthin-6-one	$C_{13}H_{12}N_2O_2$	236.2297	0	0	0	1	0.0586	-0.0051
8-hydroxylcanthin-6-one	$C_{14}H_8N_2O_2$	236.2297	0	0	0	1	0.0586	-0.0051
1-ethoxymethenyl-β-carboline	$C_{14}H_8N_2O_2$	240.2615	3	4	0	2	0.0899	0.0212
β-carboline-1-propanoic acid	$C_{14}H_{12}N_2O_2$	240.2615	3	4	0	2	0.0899	0.0212
1-methenyl-4-methoxyl-β-carboline	$C_{14}H_{12}N_2O_2$	240.2615	3	4	0	2	0.0899	0.0212
1-ethenyl-4-methoxyl-8-hydroxyl-β-carboline	$C_{14}H_{12}N_2O_2$	240.2615	3	4	0	2	0.0899	0.0212
1-ethyl-4-methoxyl-8-hydroxyl-β-carboline	$C_{14}H_{12}N_2O_2$	242.2774	3	6	0	2	0.1055	0.0368
1-methoxymethenyl-4-hydroxyl-β-carboline	$C_{14}H_{14}N_2O_2$	242.2774	2	2	0	3	0.0691	0.0004
1-(2-hydroxyl)-ethyl-4-methoxyl-β-carboline	$C_{14}H_{14}N_2O_2$	242.2774	3	6	0	2	0.1055	0.0368
8-methoxyl-1,2,3,4-Tetrahydro-1,3,4-trioxo-β-carboline	$C_{13}H_{10}N_2O_3$	244.2065	1	0	0	4	0.0484	-0.0203
3-methylcanthin-2,6-dione	$C_{12}H_8N_2O_4$	250.2566	0	0	0	0	0.0742	0.0000
5-methoxylcanthin-6-one	$C_{15}H_{10}N_2O_2$	250.2566	1	2	0	1	0.0742	0.0105
3-methylcanthin-5,6-dione	$C_{15}H_{10}N_2O_2$	250.2566	0	0	0	0	0.0742	0.0000
1-ethenyl-4,8-dimethoxyl-β-carboline		254.2884	4	6	0	2	0.1055	0.0368
1-ethenyl-4,9-dimethoxyl-β-carboline	$C_{15}H_{14}N_2O_2$	254.2884	4	6	0	2	0.1055	0.0368
1-methoxypropionyl-β-carboline	$C_{15}H_{14}N_2O_2$	254.2884	4	6	0	2	0.0899	0.0212
1-ethyl-4,8-dimethoxyl-β-carboline	$C_{15}H_{14}N_2O_2$	256.3042	4	8	0	2	0.1212	0.0525
β-carboline-1-(2-hydroxyl)-propanoic acid	$C_{14}H_{12}N_2O_3$	256.2609	3	4	0	3	0.0848	0.0161
1-(1,2-dihydroxyl)-ethyl-4-methoxyl-β-carboline	$C_{15}H_{16}N_2O_2$	258.2768	3	6	0	3	0.1004	0.0317

4-hydroxyl-5-methoxylcanthin-6-one	$C_{14}H_{14}N_2O_3$	266.2560	1	2	0	2	0.0691	0.0054
3-methyl-4-hydroxylcanthin-5,6-dione	$C_{15}H_{10}N_2O_3$	266.2560	0	0	0	1	0.0691	-0.0051
4-methoxyl-5-hydroxylcanthin-6-one	$C_{15}H_{10}N_2O_3$	266.2560	1	2	0	2	0.0691	0.0054
1-(1-hydroxyl)-ethyl-4,8-dimethoxyl-β-carboline	$C_{14}H_{12}N_2O_4$	272.3036	4	8	0	3	0.1161	0.0474
1-(2-hydroxyl)-ethyl-4,8-dimethoxyl-β-carboline	$C_{14}H_{12}N_2O_4$	272.3036	4	8	0	3	0.1161	0.0474
β-carboline-1-(1,2-dihydroxyl)-propanoic acid	$C_{15}H_{10}N_2O_3$	272.2603	3	4	0	4	0.0797	0.0110
1-methoxymethenyl-4-methoxyl-8-hydroxyl-β-carboline	$C_{15}H_{10}N_2O_3$	272.2603	3	4	0	4	0.0797	0.0110
3-methyl-4-methoxylcanthin-5,6-dione	$C_{16}H_{12}N_2O_3$	280.2829	1	2	0	1	0.0848	0.0106
4,5-dimethoxylcanthin-6-one	$C_{16}H_{12}N_2O_3$	280.2829	2	4	0	2	0.0848	0.0211
4,10-dihydroxyl-5-methoxylcanthin-6-one	$C_{15}H_{10}N_2O_4$	282.2554	1	2	0	3	0.0641	0.0004
1-(2-ethoxyl)-ethanol-4-methoxyl-β-carboline	$C_{16}H_{18}N_2O_3$	286.3305	5	10	0	3	0.1317	0.0630
1-(2-methoxyl)-ethyl-4,8-dimethoxyl-β-carboline	$C_{16}H_{18}N_2O_3$	286.3305	5	10	0	3	0.1317	0.0630
1-(1,2-dihydroxyl)-ethyl-4,8-dimethoxyl-β-carboline	$C_{15}H_{16}N_2O_4$	288.3030	4	8	0	4	0.1110	0.0423
1-(2-diethylamino)-ethyl-4-methoxyl-8-hydroxyl-β-carboline	$C_{18}H_{23}N_3O_2$	313.3995	7	15	1	2	0.1790	0.1103
1-(1-carbonyl-2-alkenyl)-methoxybutyl-4,8-dimethoxyl-β-carboline	$C_{18}H_{16}N_2O_5$	340.3354	7	8	0	5	0.1059	0.0372

Table S2. Primary network composed of fragment ions with NTAs ≥ 4*

Fragment ion (*m/z*)	Predicted formula	Compound number
101.0386	C_8H_5	5,7,10,33,49,61
103.0542	C_8H_7	5,13,52,63,64
114.0464	C_9H_6	2,11,22,30
115.0542	C_9H_7	1,3,5,6,9,11,13,15,17,18,19,20,21,22,24,28,29,30,35,36,37,41,43,52,65,67,75, kx3
117.0573	C_8H_7N	31,61,67,71
127.0417	C_9H_5N	42,44,48,61,68,72
127.0542	$C_{10}H_7$	3,4,5,6,9,11,12,19,22,23,29,39,40,59,62,63
128.0495	C_9H_6N	3,5,7,9,10,12,13,21,22,27,29,31,33,39,49,61,62,63,64,67
129.0573	C_9H_7N	13,21,34,35,40,67
129.0699	$C_{10}H_9$	2,11,22,30
130.0651	C_9H_8N	13,52,62,63,64
140.0495	$C_{10}H_6N$	1,2,3,5,6,9,11,13,19,20,21,22,24,25,28,29,30,34,35,40,41,42,44,46,48,51,52,55,61,63 ,65,67,68,69,70,72, kx3
141.0573	$C_{10}H_7N$	2,11,21,22,30,71
142.0651	$C_{10}H_8N$	1,2,3,9,11,18,20,29,31,35,47,75
145.0522	C_9H_7NO	5,31,67,71
152.0495	$C_{11}H_6N$	1,9,21,22
153.0573	$C_{11}H_7N$	4,12,21,22,23,39,60,63,67
154.0525	$C_{10}H_6N_2$	42,44,48,61,68,72
154.0651	$C_{11}H_8N$	2,3,4,5,6,9,11,12,13,17,19,21,22,23,26,28,29,30,31,34,37,39,40,59,62,63,67,75
155.0604	$C_{10}H_7N_2$	5,7,10,13,21,22,27,33,49,54,57,61,71,73,kx2
155.0730	$C_{11}H_9N$	26,28,34,40
156.0444	$C_{10}H_6NO$	5,7,10,27,57,61,67
156.0682	$C_{10}H_8N_2$	5,13,32,69
166.0651	$C_{12}H_8N$	5,13,14,41,65,69,75, kx3
167.0604	$C_{11}H_7N_2$	2,3,5,6,7,8,9,11,13,15,17,19,20,21,22,24,25,29,34,35,37,41,42,44,46,48,51,52,55,63, 65,67,68,69,70,72,75, kx3
168.0682	$C_{11}H_8N_2$	2,5,11,13,14,18,21,22,24,30,36,38,47,51,54,56,69,71,76, kx3
169.0396	$C_{10}H_5N_2O$	42,44,48,68,72
169.0522	$C_{11}H_7NO$	22,30,54,71
169.0760	$C_{11}H_9N_2$	1,3,5,7,8,13,19,21,22,24,31,32,34,35,42,43,44,47,48,54,67,68,71,72,75,76, kx3
170.0600	$C_{11}H_8NO$	1,9,14,19,31,47,67,73,76
170.0838	$C_{11}H_{10}N_2$	1,3,5,31,35,75
171.0553	$C_{10}H_7N_2O$	50,61,71,74,76,kx1
171.0679	$C_{11}H_9NO$	14,19,31,45,67
179.0604	$C_{12}H_7N_2$	1,3,4,5,7,9,10,12,21,22,23,28,51,60,76
180.0682	$C_{12}H_8N_2$	7,8,21,22,28,39,51,60
181.0760	$C_{12}H_9N_2$	2,3,4,5,6,9,11,12,13,14,15,16,17,19,22,23,26,29,37,39,40,59,62,63,67,75
182.0475	$C_{11}H_6N_2O$	42,44,48,53,61,68,72
182.0838	$C_{12}H_{10}N_2$	2,3,5,8,11,13,15,16,17,18,22,26,34,40,54,67
183.0553	$C_{11}H_7N_2O$	5,7,10,13,21,22,27,33,49,53,57,63

183.0917	$C_{12}H_{11}N_2$	3,5,13,14,22,47,54,67,69
184.0393	$C_{11}H_6NO_2$	6,19,31,67
184.0631	$C_{11}H_8N_2O$	5,32,47,50,63,64,67,73,74,75,kx2
184.0757	$C_{12}H_{10}NO$	14,31,67,69
185.0709	$C_{11}H_9N_2O$	13,19,32,34,39,42,48,63,67,69,71,74
192.0682	$C_{13}H_8N_2$	5,13,26,41,65,70,75, kx3
193.0760	$C_{13}H_9N_2$	3,5,9,13,20,24,28,38,41,47,59,65,75, kx3
195.0553	$C_{12}H_7N_2O$	14,22,30,70,71,74
195.0917	$C_{13}H_{11}N_2$	3,5,6,9,13,15,17,19,20,28,29,36,37,54
196.0631	$C_{12}H_8N_2O$	1,14,21,22,30,54,71,76
197.0709	$C_{12}H_9N_2O$	1,5,7,8,9,21,22,31,32,34,35,42,43,44,45,47,48,58,67,68,71,72,73,75,76
198.0788	$C_{12}H_{10}N_2O$	3,5,6,8,19,31,35,50,54,67
208.0631	$C_{13}H_8N_2O$	51,53,59,60,72
209.0709	$C_{13}H_9N_2O$	5,13,39,40,47,59,62,63,67,75
210.0788	$C_{13}H_{10}N_2O$	5,13,34,40,47,54,67
211.0866	$C_{13}H_{11}N_2O$	8,14,15,16,17,22,30,45,47,54,58,67,69,75,76
212.0944	$C_{13}H_{12}N_2O$	32,43,47,58
213.0659	$C_{12}H_9N_2O_2$	32,34,57,71,74
223.0866	$C_{14}H_{11}N_2O$	6,15,19,28,37,59,75
224.0580	$C_{13}H_8N_2O_2$	14,47,69,71
225.0659	$C_{13}H_9N_2O_2$	31,45,47,58,67
226.0737	$C_{13}H_{10}N_2O_2$	19,31,32,45,50,67
227.0815	$C_{13}H_{11}N_2O_2$	32,42,48,50,58,73,74
239.0815	$C_{14}H_{11}N_2O_2$	45,47,58,67,69,71,75
240.0893	$C_{14}H_{12}N_2O_2$	45,47,58,67
241.0972	$C_{14}H_{13}N_2O_2$	32,45,50,54

* : The value of NTAs was screening by using the functions written in MATLAB, in our laboratory.

Table S3. Filter references for MDF: dimer alkaloids summarized from literatures

Chemical name	Molecular formula	Molecular weight	Formula change				Mass defect	Mass defect shift(Da)
			C-	H-	N-	O-		
Dimer Alkaloids standard:	$C_{22}H_{16}N_4$	336.3960	/	/	/	/	0.1374	/
picrasidineA	$C_{27}H_{22}N_4O_3$	450.4967	5	6	0	3	0.1692	0.0004
picrasidinesG	$C_{28}H_{24}N_4O_2$	448.5242	6	8	0	2	0.1899	0.0211
picrasidinesF	$C_{29}H_{25}N_4O_3$	478.5900	8	12	0	4	0.2111	0.0423
picrasidinesH	$C_{28}H_{24}N_4O_4$	480.5230	6	8	0	4	0.1848	0.0160
picrasidinesM	$C_{29}H_{22}N_4O_4$	490.5181	7	6	0	4	0.1641	0.0211
picrasidinesN	$C_{29}H_{22}N_4O_4$	490.5181	7	6	0	4	0.1641	0.0211
picrasidineC	$C_{29}H_{26}N_4O_4$	494.5498	7	10	0	4	0.1954	0.0266
picrasidinesT	$C_{29}H_{26}N_4O_4$	494.5498	7	10	0	4	0.1954	0.0266
picrasidinesS	$C_{30}H_{28}N_4O_4$	508.5767	8	12	0	4	0.2111	0.0423
picrasidinesU	$C_{30}H_{24}N_4O_5$	520.5444	8	8	0	5	0.1747	0.0317
picrasidinesR	$C_{30}H_{26}N_4O_6$	538.5596	8	10	0	6	0.1852	0.0164

For the dimer alkaloids, based on the information of Supplementary Table S2, the minimum and maximum values of mass defects were calculated as 0.1641 and 0.2111 Da, corresponding to the formula of $C_{29}H_{22}N_4O_4$ and $C_{30}H_{28}N_4O_4$, respectively. Therefore, the filter was set as $C_{29.5}H_{25}N_4O_4$±23.5 mDa over the mass range of 340-700 Da. The filtered chromatogram was shown in Supplementary Fig S2 and its noise level ($1.5 \times 10e^6$ counts per second, cps) was 21% of that of the original chromatogram ($7 \times 10\ e^6$ cps) (Fig. 2A). After excluding the irrelevant ions by MDF, a total of 33 dimer candidates (Table 1) were detected in the filtered TIC profiles.

Table S4. The primary network of dimer alkaloids

Ion fragments(m/z)	Predicted formula	Dimer alkaloid candidatess
169.076	$C_{11}H_9N_2$	77,78,80,90,91,92,93,95,97,105
181.076	$C_{12}H_9N_2$	77,78,80,95,97,107,z2
182.0838	$C_{12}H_{10}N_2$	77,79,84,85,91,95,97
193.076	$C_{13}H_9N_2$	84,91,95,97,106,107,109
195.0917	$C_{13}H_{11}N_2$	77,78,79,80,84,91,93,95,97
197.0709	$C_{12}H_9N_2O$	82,86,87,90,93,95,98,100,103,104,109
199.0866	$C_{12}H_{11}N_2O$	81,82,90,103,104,109
205.076	$C_{14}H_9N_2$	79,85,90,97,107
206.0838	$C_{14}H_{10}N_2$	77,78,80,90,91,95,97
207.0917	$C_{14}H_{11}N_2$	77,78,80,84,91,95,97
208.0995	$C_{14}H_{12}N_2$	84,91,95,97
209.0709	$C_{13}H_9N_2O$	81,106,107,z2
211.0866	$C_{13}H_{11}N_2O$	77,79,51,82,85,86,87,90,93,95,97,98,100,103,104,107
212.0944	$C_{13}H_{12}N_2O$	82,86,87,90,93,95,96,97,98,100,103,104,109
213.0659	$C_{12}H_9N_2O_2$	81,83,84,86,87,88,89
213.1022	$C_{13}H_{13}N_2O$	86,87,90,105
221.0709	$C_{14}H_9N_2O$	90,95,97,100,103,106,107
222.0788	$C_{14}H_{10}N_2O$	77,86,87,90,93,95,97,100,103
223.0866	$C_{14}H_{11}N_2O$	81,82,86,87,90,93,95,97,98,100,103,107,z2
224.0944	$C_{14}H_{12}N_2O$	81,82,90,95,97,100,103,104,105,z2
225.1022	$C_{14}H_{13}N_2O$	77,82,86,87,96,97,98,99,100,103,104,105,106
226.0737	$C_{13}H_{10}N_2O_2$	78,80,82,94
227.0815	$C_{13}H_{11}N_2O_2$	81,82,83,84,86,87,88,89,91,98,101,104,108,109
228.0893	$C_{13}H_{12}N_2O_2$	81,83,84,86,87,88,89,91,98,101
235.0866	$C_{15}H_{11}N_2O$	82,85,90,91,95,97,98,100,101,103,104,106,107,109
236.0944	$C_{15}H_{12}N_2O$	82,90,93,95,97,100,103,104
237.1022	$C_{15}H_{13}N_2O$	82,86,87,93,95,97,98,100,103,104
238.0737	$C_{14}H_{10}N_2O_2$	81,83,84,86,87,88,89,106
238.1101	$C_{15}H_{14}N_2O$	82,86,87,93,98
239.0815	$C_{14}H_{11}N_2O_2$	81,83,84,86,87,88,89,107,109
240.0893	$C_{14}H_{12}N_2O_2$	79,82,91,92,96,102,109,z1
241.0972	$C_{14}H_{13}N_2O_2$	78,79,80,82,83,86,87,88,89,91,92,94,98,101,104,106
242.105	$C_{14}H_{14}N_2O_2$	82,88,89,91,96,98,101,104
249.1022	$C_{16}H_{13}N_2O$	82,95,97,100,103,104
250.1101	$C_{16}H_{14}N_2O$	82,95,97,100,103,104
251.1179	$C_{16}H_{15}N_2O$	82,93,95,103
252.0893	$C_{15}H1_2N_2O_2$	78,80,82,86,88,89,91,98,101,104
253.0972	$C_{15}H_{13}N_2O_2$	81,82,83,84,86,87,88,89,98,101,104,106
254.105	$C_{15}H_{14}N_2O_2$	82,86,104,109
255.1128	$C_{15}H_{15}N_2O_2$	77,78,79,80,82,91,96,98,102,104,105,z1

265.0972	$C_{16}H_{13}N_2O_2$	79,82,94,104
267.1128	$C_{16}H_{15}N_2O_2$	82,86,88,89,91,98,101,104
268.1206	$C_{16}H_{16}N_2O_2$	88,89,98,101
283.1104	$C_{19}H_{13}N_3$	82,91,92,94
388.1319	$C_{25}H_{16}N_4O$	85,90,95,97,107
403.1553	$C_{26}H_{19}N_4O$	85,90,95,97,105,107
418.1424	$C_{26}H_{18}N_4O_2$	91,92,100,105,106
433.1659	$C_{27}H_{21}N_4O_2$	79,92,100,103,105,106

Table S5. Detected and reported isomers of dimer alkaloids in *P. quassioides*

$[M+H]^+$ (m/z)	Detected Compound No.	Amount of isomer detected /reported
423	77	1/1
453	78	1/1
483	79	1/0
467	80	1/1
481	83	1/1
521	106	1/1
491	107	1/2
537	108	1/0
545	109	1/0
435	84,85	2/0
525	94,96	2/0
509	101,104	2/1
539	102,105	2/1
451	81,92,99	3/1
495	82,88,89	3/2
465	86,87,90	3/0
479	98,100,103	3/1
449	91,93,95,97	3/1

Table S6 Identified dimer alkaloids from the extract of *P. quassioides*

No.	Rt (min)	[M+H]+ (m/z)	Tolerance (mDa)	Predicted formula	Assignment	Remark
77	19.449	423.1800	0.18	$C_{26}H_{22}N_4O_2$	Quassidine C	IP, 31
78	24.560	453.1911	-0.31	$C_{27}H_{24}N_4O_3$	Quassidine B	IP, 31
79	33.741	483.2021	0.55	$C_{28}H_{26}N_4O_4$	Unknown	
80	34.097	467.2066	-0.18	$C_{28}H_{26}N_4O_3$	Quassidine D	IP, 31
81	48.843	451.1759	0.54	$C_{27}H_{22}N_4O_3$	unknown	
82	51.481	495.2014	-0.10	$C_{29}H_{26}N_4O_4$	Isopicrasidines T	IE, N
83	52.194	481.1865	0.50	$C_{28}H_{24}N_4O_4$	Picrasidines H	IP, 32
84	54.050	435.1809	0.66	$C_{27}H_{22}N_4O_2$	unknown	
85	58.174	435.1811	0.44	$C_{27}H_{22}N_4O_2$	unknown	
86	59.922	465.1912	0.87	$C_{28}H_{24}N_4O_3$	unknown(optical isomer)	
87	60.672	465.1919	0.25	$C_{28}H_{24}N_4O_3$	unknown(optical isomer)	
88	64.311	495.2022	-0.86	$C_{29}H_{26}N_4O_4$	unknown	
89	65.517	495.2021	-0.76	$C_{29}H_{26}N_4O_4$	unknown	
90	66.910	465.1914	0.72	$C_{28}H_{24}N_4O_3$	unknown	
91	66.973	449.1966	0.61	$C_{28}H_{24}N_4O_2$	unknown	
92	67.855	451.1760	0.42	$C_{27}H_{22}N_4O_3$	Picrasidines A	IP, 20
93	69.057	449.1969	0.35	$C_{28}H_{24}N_4O_2$	Isopicrasidines G1	IE, N
94	72.001	525.2126	0.65	$C_{30}H_{28}N_4O_5$	unknown	
95	72.022	449.1966	0.61	$C_{28}H_{24}N_4O_2$	Picrasidines G	IP, 33
96	73.394	525.2129	0.35	$C_{30}H_{28}N_4O_5$	unknown	
97	73.978	449.1964	-0.48	$C_{28}H_{24}N_4O_2$	Isopicrasidines G2	IE, N
98	74.027	479.2075	0.76	$C_{29}H_{26}N_4O_3$	Picrasidines F	IP, 34
99	75.935	451.1758	0.66	$C_{27}H_{22}N_4O_3$	unknown	
100	80.957	479.2080	-0.26	$C_{29}H_{26}N_4O_3$	unknown	
101	81.554	509.2180	1.34	$C_{30}H_{28}N_4O_4$	Picrasidines S	IP, 33
102	82.007	539.1920	-0.79	$C_{30}H_{26}N_4O_6$	Picrasidine R	IP, 32
103	82.147	479.2077	0.09	$C_{29}H_{26}N_4O_3$	unknown	
104	87.621	509.2182	0.66	$C_{30}H_{28}N_4O_4$	isopicrasidines S	IE, N
105	94.599	539.1924	0.10	$C_{30}H_{26}N_4O_6$	unknown	
106	96.318	521.1821	-0.12	$C_{30}H_{24}N_4O_5$	Picrasidines U	IP
107	96.565	491.1721	-0.70	$C_{29}H_{22}N_4O_4$	unknown	
108	96.675	537.1774	-0.52	$C_{30}H_{24}N_4O_6$	unknown	
109	97.275	545.1820	-0.05	$C_{32}H_{24}N_4O_5$	unknown	
Kx4	32.874	491.1711	0.33	$C_{29}H_{22}N_4O_4$	Picrasidines M	IE, 35
Kx5	50.055	491.1704	1.00	$C_{29}H_{22}N_4O_4$	Picrasidines N	IE

IE: identified by the enhanced network, IP: identified by the primary network, N: the new structure; [1-6]: the identified structure was validated by the information of indicated literature.

Reference

15. Z. Lai, Master Thesis, Guangzhou University of Chinese Medicine,Guangzhou, China, 2011.

W. Jiao, H. Gao, C. Li, F. Zhao, R. Jiang, Y. Wang, G. Zhou, X. Yao, Quassidines A-D, bis- -carboline alkaloids from the stems of *Picrasma quassioides*, J. Nat. Prod. 73 (2010) 167-171.

T. Ohmoto, K. Koike, tudies on the Alkaloids from *Picrasma quassioides* Bennet.VII. structures of β-carboline dimer alkaloids, picrasidines-H and –R, Chem Pharm Bull. 34 (1986) 2090-2093.

T. Ohmoto, K. Koike, Studies on the alkaloids from *Picrasma quassioides* Bennet. IX. structures of two β-carboline dimeric alkaloids, picrasidines-G and -S, Chem Pharm Bull. 35 (1987) 3305-3308.
T. Ohmoto, K. Koike, Studies on the alkaloids of *Picrasma quassioides* Bennet. VIII. x-ray crystal structure analysis of picrasidine-F, Chem Pharm Bull. 34 (1986) 3228-3236.
T. Ohmoto, K. Koike, Studies on the alkaloids from *Picrasma quassioides* Bennet. V. structures of picrasidines L, M, and P, Chem Pharm Bull. 33 (1985) 3847-3851.

Supplementary Figure legends

Supplementary Figure 1. Cleavage pathways of compounds **kx1, 61, kx2, kx3, 5** and **13.**

Supplementary Figure 2. Corresponding filtered chromatogram of dimer alkaloids by mass defect filter.

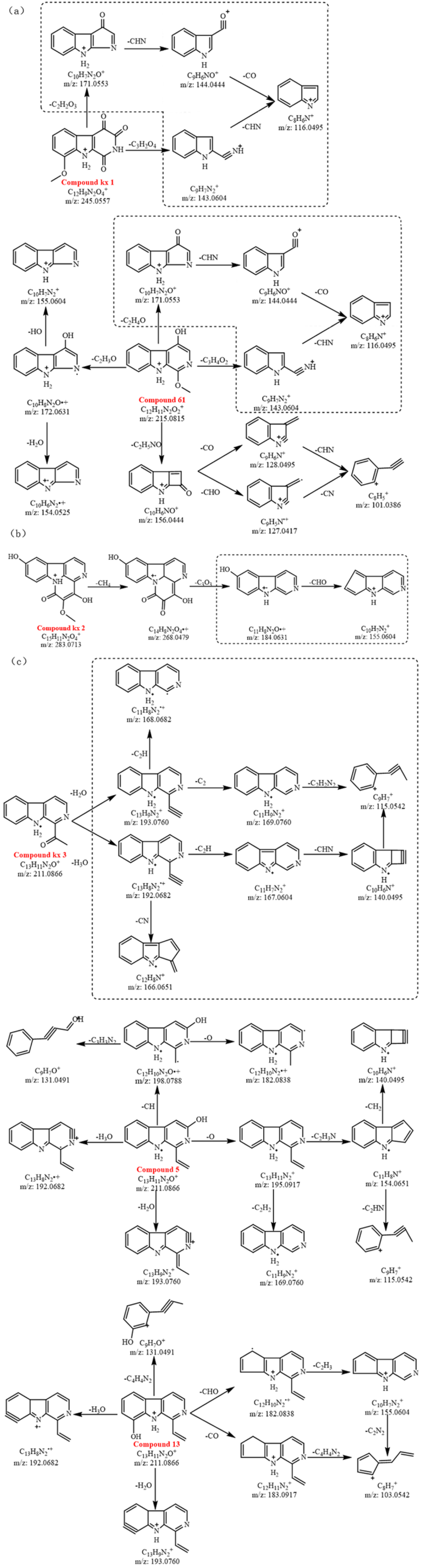

(a)
-CHN
$C_{10}H_7N_2O^+$
m/z: 171.0553
$C_9H_6NO^+$
m/z: 144.0444
-CO
$C_8H_6N^+$
m/z: 116.0495
$-C_2H_2O_3$
-CHN
Compound kx 1
$C_{12}H_9N_2O_4^+$
m/z: 245.0557
$-C_3H_2O_4$
$C_9H_7N_2^+$
m/z: 143.0604
$C_{10}H_7N_2^+$
m/z: 155.0604
-HO
$C_{10}H_7N_2O^+$
m/z: 171.0553
-CHN
$C_9H_6NO^+$
m/z: 144.0444
-CO
$C_8H_6N^+$
m/z: 116.0495
$-C_2H_4O$
-CHN
$-C_2H_3O$
$-C_3H_4O_2$
$C_{10}H_8N_2O^{•+}$
m/z: 172.0631
Compound 61
$C_{12}H_{11}N_2O_2^+$
m/z: 215.0815
$C_9H_7N_2^+$
m/z: 143.0604
$-H_2O$
$-C_2H_5NO$
-CO
$C_9H_6N^+$
m/z: 128.0495
-CHN
-CHO
-CN
$C_{10}H_6N_2^{•+}$
m/z: 154.0525
$C_{10}H_6NO^+$
m/z: 156.0444
$C_9H_5N^{•+}$
m/z: 127.0417
$C_8H_5^+$
m/z: 101.0386
(b)
$-CH_4$
$-C_3O_3$
-CHO
Compound kx 2
$C_{15}H_{11}N_2O_4^+$
m/z: 283.0713
$C_{14}H_8N_2O_4^{•+}$
m/z: 268.0479
$C_{11}H_8N_2O^{•+}$
m/z: 184.0631
$C_{10}H_7N_2^+$
m/z: 155.0604
(c)
$C_{11}H_8N_2^{•+}$
m/z: 168.0682
$-C_2H$
$-C_2$
$-C_2H_2N_2$
$-H_2O$
$C_{13}H_9N_2^+$
m/z: 193.0760
$C_{11}H_9N_2^+$
m/z: 169.0760
$C_9H_7^+$
m/z: 115.0542
Compound kx 3
$C_{13}H_{11}N_2O^+$
m/z: 211.0866
$-H_2O$
$-C_2H$
-CHN
$C_{13}H_8N_2^{•+}$
m/z: 192.0682
$C_{11}H_7N_2^+$
m/z: 167.0604
$C_{10}H_6N^+$
m/z: 140.0495
-CN
$C_{12}H_8N^+$
m/z: 166.0651
$-C_3H_3N_2$
-O
$C_9H_7O^+$
m/z: 131.0491
$C_{12}H_{10}N_2O^{•+}$
m/z: 198.0788
$C_{12}H_{10}N_2^{•+}$
m/z: 182.0838
$C_{10}H_6N^+$
m/z: 140.0495
-CH
$-CH_2$
$-H_2O$
-O
$-C_2H_3N$
$C_{13}H_8N_2^{•+}$
m/z: 192.0682
Compound 5
$C_{13}H_{11}N_2O^+$
m/z: 211.0866
$C_{13}H_{11}N_2^+$
m/z: 195.0917
$C_{11}H_8N^+$
m/z: 154.0651
$-H_2O$
$-C_2H_2$
$-C_2HN$
$C_{13}H_9N_2^+$
m/z: 193.0760
$C_{11}H_9N_2^+$
m/z: 169.0760
$C_9H_7^+$
m/z: 115.0542
HO
$C_9H_7O^+$
m/z: 131.0491
$-C_4H_4N_2$
-CHO
$-C_2H_3$
$-H_2O$
$C_{12}H_{10}N_2^{•+}$
m/z: 182.0838
$C_{10}H_7N_2^+$
m/z: 155.0604
$C_{13}H_8N_2^{•+}$
m/z: 192.0682
Compound 13
$C_{13}H_{11}N_2O^+$
m/z: 211.0866
-CO
$-C_2N_2$
$-C_4H_4N_2$
$C_{12}H_{11}N_2^+$
m/z: 183.0917
$C_8H_7^+$
m/z: 103.0542
$-H_2O$
$C_{13}H_9N_2^+$
m/z: 193.0760

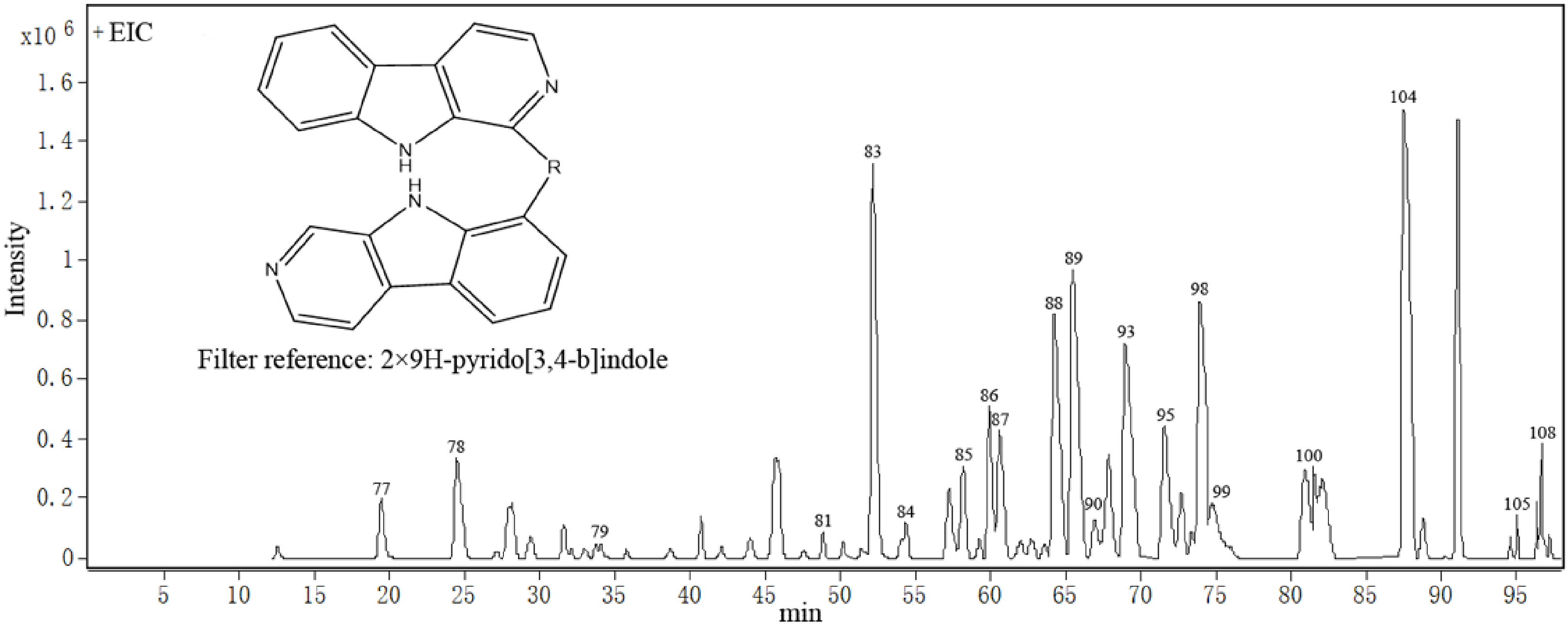
x10 6
+EIC
Filter reference: 2×9H-pyrido[3,4-b]indole
Intensity
min
N
H
R
1.6
1.4
1.2
1
0.8
0.6
0.4
0.2
0
5
10
15
20
25
30
35
40
45
50
55
60
65
70
75
80
85
90
95
77
78
79
81
83
84
85
86
87
88
89
90
93
95
98
99
100
104
105
108

Supplementary materials

The following code is the main code for selecting bridging ions and establish the MS network. "%" refers to the explanation of the code.

```
m = xlsread('zhongyao1.xlsx');        % read the data from excel
a=m(:,1);                             % list a into the first column
b=m(:,2);                             % list b into the second column
a = a( ~ isnan(a));                   % delete the value of nan(a)
b = b( ~ isnan(b));                   % delete the value of nan(b)
c = [a b];                            % obtain a matrix
for n=1:max(a)                        % loop from n equal to 1 until maximize n;
    d = find(c(:,1)==n);              % find the position of the data (which equals to n) from column 1
    e = c(d,2);                       % obtain the data below into a new matrix
for i=1:length(e)                     % i equals to the length from 1 to e (e represents the serial numbers of
                                        the precursor ions which fragments' NTAs equals to n)
     for j =1:length(b)               % j equals to the length from 1 to b (b represents the serial numbers
                                        of all the precursor ions)
            if b(j)==e(i)
               b(j)=[];               % delete all the data of b(j)
               break;
            end
        end
    end
    if length(unique(b))<60           % loop until the length of unique(b) is less than 60
        break;
    end
end
disp(n);
disp(max(a));
```

The design surface of this program draws as follows:

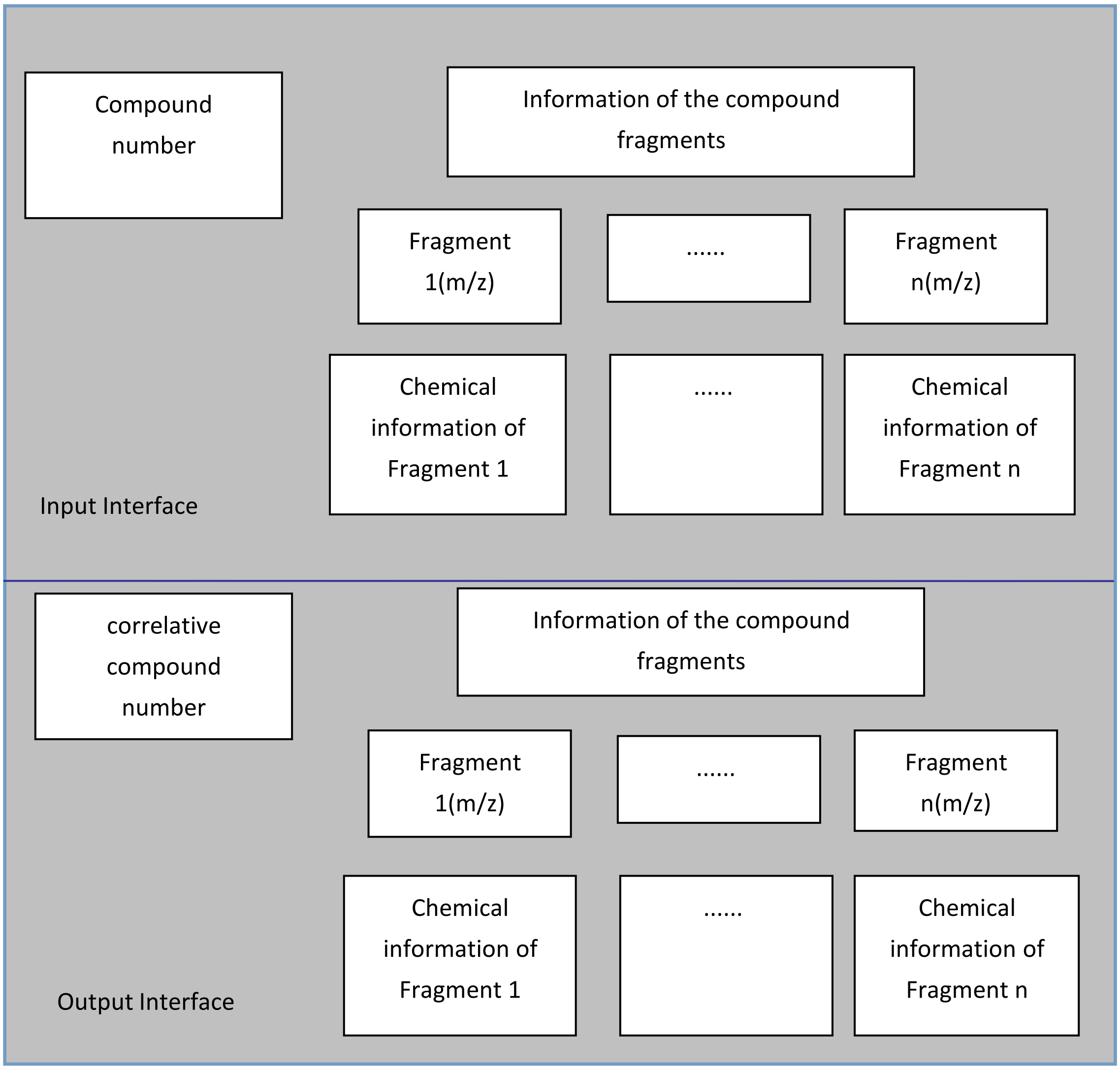

Supplement: Supplementary file 1 — Supplementary information [file 41598_2017_13106_MOESM1_ESM.pdf]
